# Simultaneous DOTATATE uptake in three non-neuroendocrine pathologies: Follicular lymphoma, Crohn’s disease, and vertebral hemangioma

**DOI:** 10.1016/j.radcr.2026.08.007

**Published:** 2026-08-30

**Authors:** Kameel Khabaz, Jonathan W. Said, Jason Chiang

**Affiliations:** a David Geffen School of Medicine, UCLA, Los Angeles, CA; b Department of Pathology and Laboratory Medicine, University of California, Los Angeles, CA; c Department of Radiological Sciences, University of California, Los Angeles, CA

**Keywords:** DOTATATE, Follicular lymphoma, Neuroendocrine tumors, Case report, Crohn’s disease, Vertebral hemangioma

## Abstract

Gallium-68 (⁶⁸Ga) DOTATATE positron emission tomography/computed tomography (PET/CT) has emerged as the gold standard imaging modality for neuroendocrine tumors (NETs). However, false-positive uptake in non-NET pathologies can be a diagnostic dilemma. The authors report a case of a 70-year-old woman presenting with recurrent small bowel obstructions, chronic diarrhea, and progressive weight loss. CT imaging demonstrated high-grade small bowel obstruction with marked bowel wall thickening and confluent mesenteric and retroperitoneal lymphadenopathy that had progressively increased over the prior year, raising clinical suspicion for NET. DOTATATE PET/CT revealed elevated uptake in the terminal ileum, mesenteric and retroperitoneal lymph nodes, and L4 vertebral body, a distribution that was suspicious for metastatic small bowel NET. However, tissue confirmation and correlation with anatomic imaging established 3 concurrent non-NET diagnoses: grade 3A follicular lymphoma, Crohn's disease, and a benign vertebral hemangioma. Simultaneous DOTATATE uptake in multiple distinct entities represents a diagnostic challenge requiring careful correlation of metabolic, anatomic, clinical, and pathologic data.

## Introduction

⁶⁸Ga-DOTATATE PET/CT has emerged as the imaging standard for characterizing neuroendocrine tumors (NETs), offering superior sensitivity and specificity compared to other imaging modalities [1]. DOTATATE is a radiolabeled somatostatin analog that localizes to tissues expressing the somatostatin receptor (SSTR), with gallium-68 serving as a positron-emitting radionucleotide that enables high-resolution PET imaging [2]. While ⁶⁸Ga-DOTATATE PET/CT is primarily indicated for imaging NETs, SSTR can also be expressed by various non-neuroendocrine tissues [2].

A growing body of literature has reported false-positive DOTATATE uptake in other malignancies, including non-Hodgkin lymphoma, breast cancer, thyroid neoplasms, renal cell carcinoma, and pancreatic ductal adenocarcinoma [[3], [4], [5], [6]]. Benign conditions demonstrating DOTATATE avidity include inflammation, reactive lymph nodes, granulomatous disease, and uterine fibroids. In addition, normal physiologic variants, such as focal DOTATATE uptake in the pancreatic uncinate process, can also mimic pathologic processes [2,7].

Misinterpretation of false-positive findings can result in critical staging errors that may misinform treatment planning. As such, visualization of DOTATATE positive findings must be integrated with clinical history, conventional imaging findings, and histopathologic confirmation when necessary [2]. This case report describes a 70-year-old female presenting with recurrent small bowel obstructions and chronic diarrhea who was initially evaluated with ^68^Ga-DOTATATE PET/CT to identify NET and was found to have 3 conditions representing false-positive findings: follicular lymphoma, Crohn’s disease, and a vertebral hemangioma.

## Case presentation

A 70-year-old female patient with a history of hypertension, hyperlipidemia, and prior pelvic surgery presented with a multiyear history of recurrent episodes of small bowel obstruction, most of which resolved spontaneously within 1-2 days. Following a diagnostic laparoscopy with lysis of adhesions, the obstructions were initially attributed to postoperative adhesions from her prior pelvic surgeries.

However, over several months, her clinical presentation continued to evolve. She developed chronic diarrhea, recurrent abdominal pain, and progressive weight loss that significantly impacted her quality of life. CT angiography demonstrated a high-grade small bowel obstruction with a transition point in the lower abdomen. However, imaging also revealed marked wall thickening and hyperemia of the jejunum and ileum, as well as confluent mesenteric and retroperitoneal lymphadenopathy and mesenteric edema that had progressively increased in size compared to the year prior. While the small bowel obstruction self-resolved, the constellation of findings created a clinical picture highly suggestive of a NET, leading the team to order a ^68^Ga-DOTATATE PET/CT scan (Siemens Multidetector, Siemens, Munich, Germany).

**Fig. 1:**
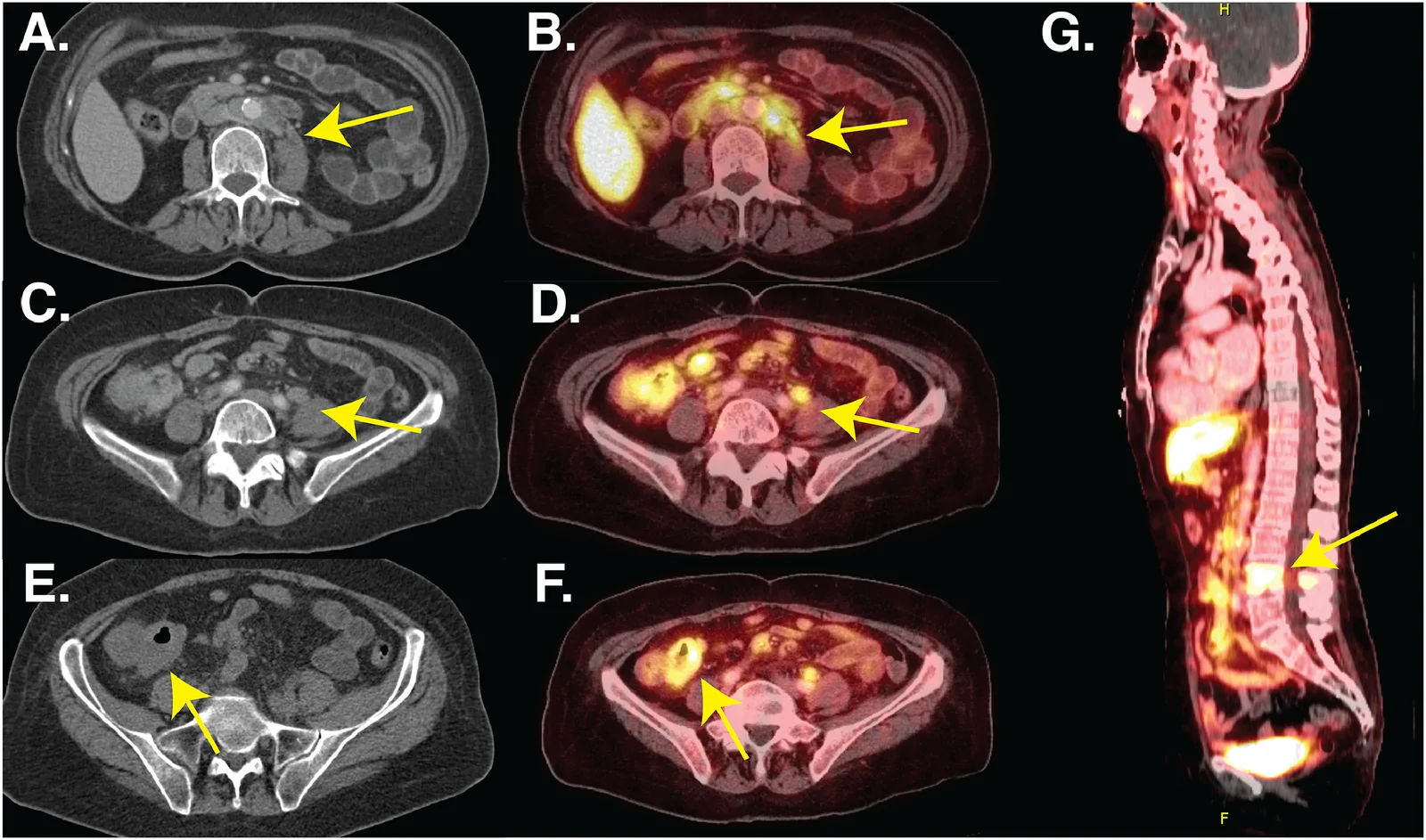
^68^Ga-DOTATATE PET/CT Findings. Axial CT (A, C, E) with fused PET/CT (B, D, F) and sagittal projection of PET/CT (G). (A, B) Left periaortic lymphadenopathy measuring up to 21 mm with moderate DOTATATE activity (SUV_max_ 6.3, yellow arrow). (C, D) DOTATATE-avid left common iliac lymph node (SUV_max_ 5.1, yellow arrow), representing biopsy-confirmed follicular lymphoma. (E, F) Terminal ileal wall thickening with moderate DOTATATE uptake (SUV_max_ 6.3, yellow arrow), representing active Crohn's disease. (G) Sagittal projection demonstrating anatomically distributed DOTATATE uptake, including L4 vertebral body with heterogeneous moderate DOTATATE activity (SUV_max_ 7.7, yellow arrow).

^68^Ga-DOTATATE PET/CT appeared to confirm the clinical suspicion for metastatic NET (Fig. 1). The scan demonstrated widespread elevated DOTATATE uptake in 3 distinct anatomic compartments. Multistation lymphadenopathy was identified, including conglomerate left periaortic nodes measuring up to 21 mm (SUV_max_ 6.3, Fig. 1A and B), a left common iliac lymph node measuring 17 × 12 mm (SUV_max_ 5.1, Fig. 1C and D), and additional enlarged nodes involving the mesentery, pericaval, and bilateral cervical chains. The terminal ileum demonstrated bowel wall thickening measuring up to 10 mm with moderate DOTATATE uptake (SUV_max_ 6.3; Fig. 1E and F). The L4 vertebral body showed heterogeneous DOTATATE activity (SUV_max_ 7.7; Fig. 1G). This distribution of uptake involving bowel, lymph nodes, and bone raised concern for metastatic small bowel NET.

**Fig. 2:**
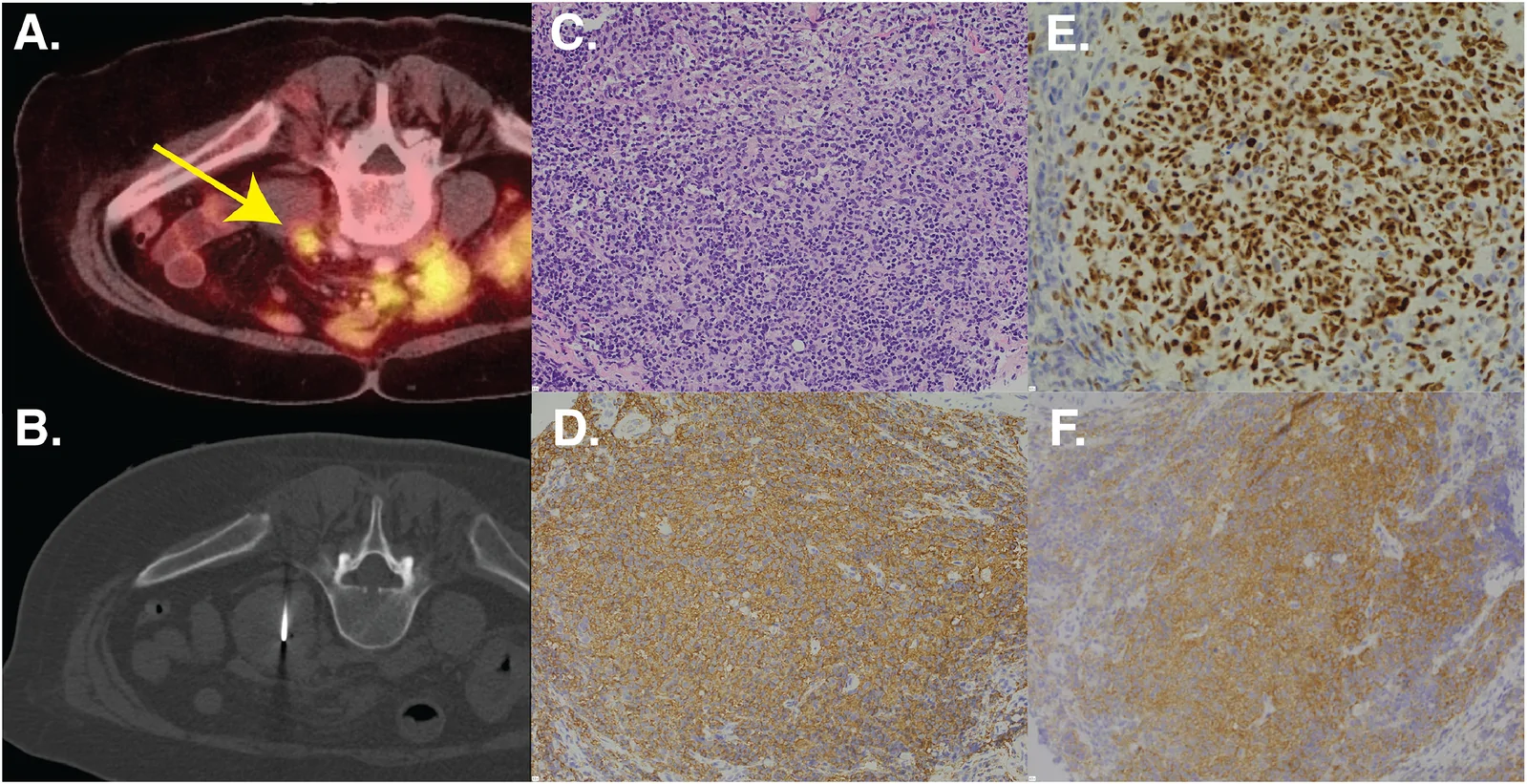
Biopsy of DOTATATE-avid lymph node. (A) Fused PET/CT shows DOTATATE-avid left common iliac lymph node (yellow arrow). (B) Axial CT image obtained during CT-guided core needle biopsy demonstrates the 18-gauge biopsy needle (arrow) within a left common iliac lymph node measuring 13 × 10 mm. (C) H&E staining shows neoplastic follicles with centrocytes and centroblasts. Immunohistochemistry illustrates positivity for CD20 (D), BCL6 (E), and CD10 (F). The immunophenotype (CD10+, CD19+, CD20+, PAX5+, BCL2+, BCL6+, and Ki-proliferation index ∼20%) confirmed grade 3A follicular lymphoma.

However, definitive diagnosis required tissue confirmation, prompting image-guided biopsy of a DOTATATE-avid left common iliac lymph node (Fig. 2A and B). Immunohistochemistry demonstrated B-cell proliferation with positivity for CD10, CD19, CD20, PAX5, BCL2, and BCL6 with kappa light chain restriction and a Ki-67 proliferation index of approximately 20%, establishing the diagnosis of grade 3A follicular lymphoma rather than NET (Fig. 2C-F). Grade 3A follicular lymphoma is a histologic subtype that occupies a controversial intermediate position between indolent low-grade FL and aggressive grade 3B disease, with implications for management [8]. The patient was classified as having high-risk follicular lymphoma with a Follicular Lymphoma International Prognostic Index (FLIPI) score of 4-5, based on her age, anemia, stage IV disease, and involvement of more than 4 nodal areas.

**Fig. 3:**
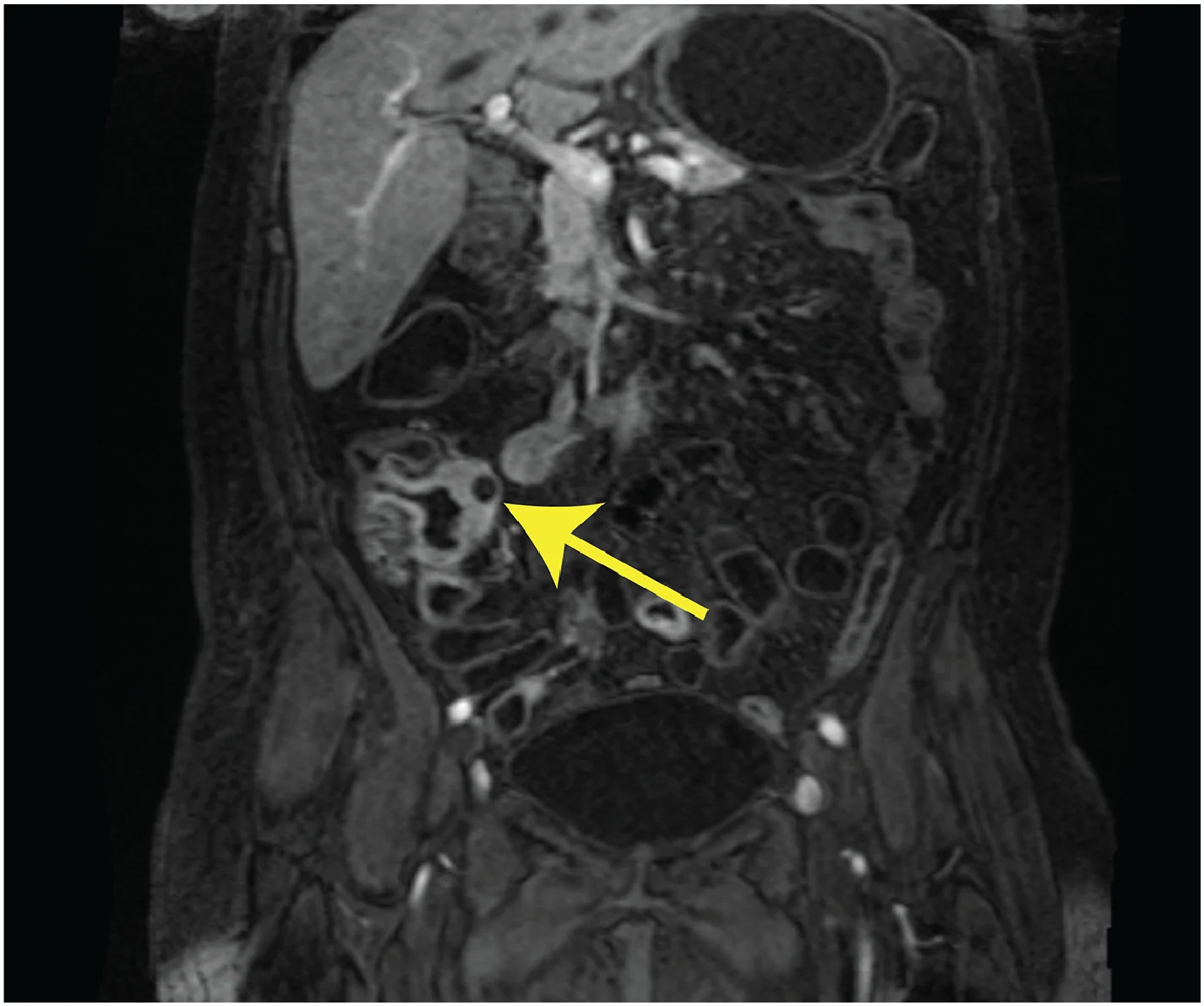
MR enterography of terminal ileum. Coronal T1-weighted contrast-enhanced MR enterography image demonstrates severe wall thickening of the terminal ileum with asymmetric mural enhancement and ulcerations (yellow arrow).

Review of prior diagnostic workup revealed additional findings. The patient had undergone colonoscopy 2 months prior revealing deep ulcerations and cobblestoning in the terminal ileum. Histopathologic evaluation demonstrated severe ileitis with ulceration and granulation tissue. Evaluation for intestinal tuberculosis was negative, including a negative interferon-γ release assay and repeat ileal biopsy showing a negative acid-fast bacilli stain. MR enterography had corroborated multifocal small bowel inflammation involving the ileum and jejunum (Fig. 3). These findings established active Crohn’s disease as the etiology of the bowel wall thickening and DOTATATE uptake in the terminal ileum. Notably, serologic testing for IBD-associated antibodies (including perinuclear antineutrophil cytoplasmic antibody, anti-*Saccharomyces cerevisiae* antibodies, etc.) had returned negative, highlighting the atypical nature of her presentation and the limitations of serologic testing in establishing this diagnosis. Furthermore, careful review of prior imaging revealed that the L4 vertebral lesion demonstrating DOTATATE activity appeared hyperdense with severe degenerative changes and had been stable for over 18 months; this was most consistent with a benign vertebral hemangioma and excluded metastatic disease.

**Fig. 4:**
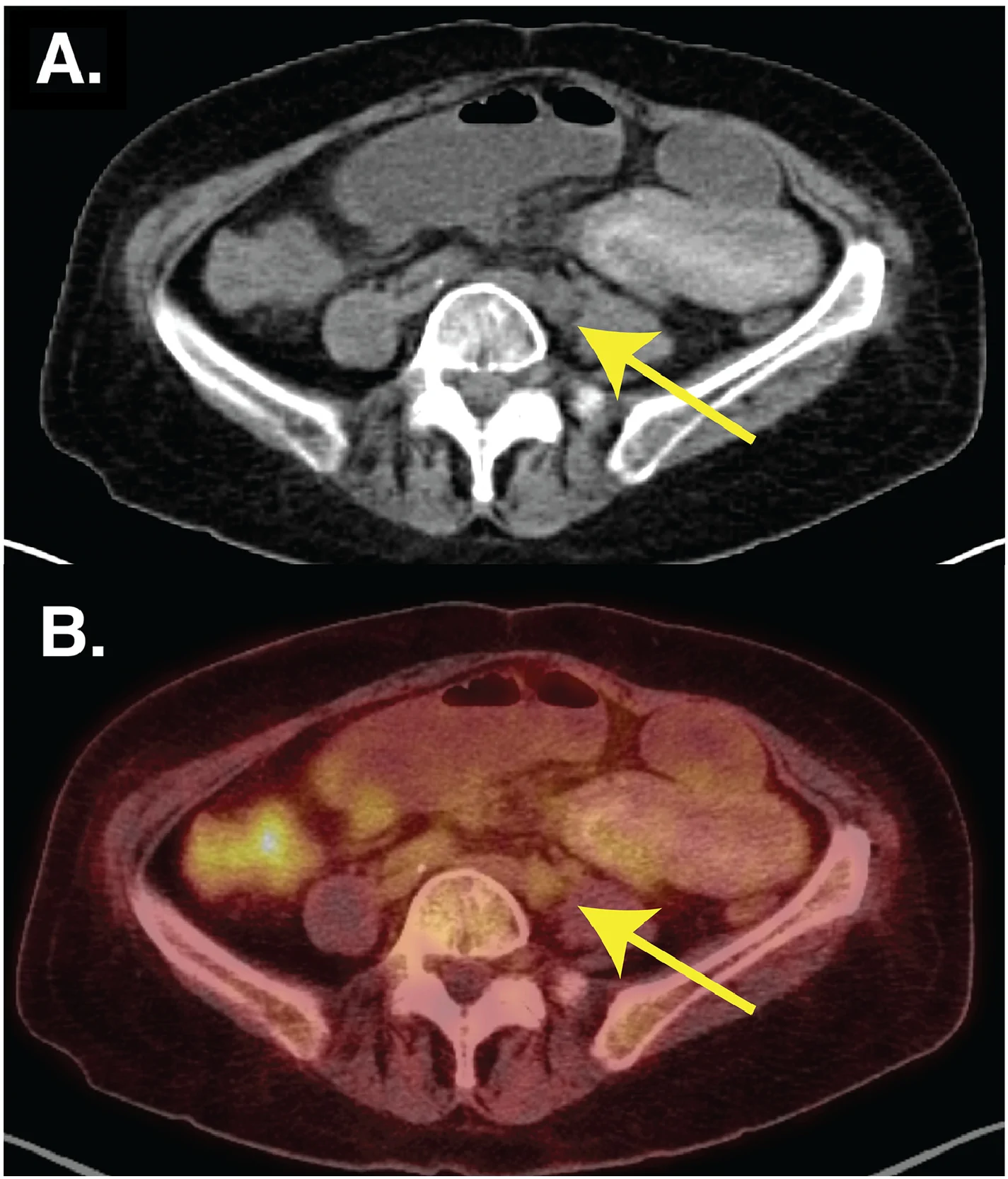
Resolution of biopsied DOTATATE-avid lymph node. CT (A) and fused PET/CT (B) show resolution of the DOTATATE-avid left common iliac lymph node (yellow arrow).

In summary, this patient’s ^68^Ga-DOTATATE PET/CT scan, obtained to evaluate for clinically suspected NET, revealed 3 distinct pathologic processes. Each process demonstrated somatostatin receptor expression: (1) grade 3A follicular lymphoma involving mesenteric and retroperitoneal lymph nodes; (2) active Crohn's disease with terminal ileal involvement; and (3) a benign vertebral hemangioma of the L4 vertebral body. Despite the compelling clinical presentation and widespread DOTATATE uptake, none of these findings represented neuroendocrine tumor. Rituximab monotherapy was initiated for follicular lymphoma with a planned course of 4 doses, with resolution of the left iliac lymph node on follow-up PET/CT (Fig. 4A and B).

## Discussion

^68^Ga-DOTATATE PET/CT has emerged as the gold standard functional imaging modality for NETs, exploiting the high expression of SSTRs on neuroendocrine tumor cells [2]. This technique provides superior sensitivity and specificity compared to conventional anatomic imaging and older scintigraphy-based methods, with a recent meta-analysis reporting sensitivity of 90.9% and specificity of 90.6% for detecting gastroenteropancreatic NETs [9]. Following FDA approval in 2016, ^68^Ga-DOTATATE PET/CT has been widely adopted for detection of occult primary tumors and evaluation of metastatic disease, noted to alter clinical management in over one-third of patients [[10], [11], [12]]. While DOTATATE imaging demonstrates excellent diagnostic performance, somatostatin receptor expression is not exclusive to neuroendocrine tumors, and false-positive uptake in non-NET pathologies is increasingly becoming recognized in the literature [2,3,6,7]. Misinterpretation of false-positive findings in non-NET pathologies can lead to critical errors in disease staging, potentially misclassifying locoregional disease as metastatic and inappropriately directing patients toward palliative rather than curative therapy. The authors present a 70-year-old female with simultaneous DOTATATE uptake in 3 anatomically separate pathologic processes: Crohn's disease, follicular lymphoma, and vertebral hemangioma. This case illustrates the importance of recognizing non-NET causes of DOTATATE uptake and the necessity of tissue diagnosis when clinical or imaging features are discordant.

While somatostatin receptor expression is classically associated with neuroendocrine tumors, its presence in lymphoproliferative disorders has been recognized since early autoradiography studies demonstrated SSTR expression in up to 90% of B-cell non-Hodgkin lymphomas [13]. Subsequent studies confirmed that various types of lymphomas can be visualized using somatostatin receptor imaging [14,15]. Despite this known expression, DOTATATE-avid follicular lymphoma remains exceedingly rare, documented only in isolated case reports [5,16,17]. This is clinically significant because follicular lymphoma is typically evaluated with FDG-PET/CT, not DOTATATE imaging, making DOTATATE-avid lymphoma an unexpected mimic of NET [18]. Follicular lymphoma typically presents with painless lymphadenopathy and is characterized by a waxing and waning clinical course, which was noted in the patient’s serial imaging. The diagnosis requires tissue confirmation via excisional or core needle biopsy, with immunohistochemistry demonstrating characteristic B-cell and germinal center markers. In the case, image-guided biopsy of the DOTATATE-avid lymph node confirmed grade 3A follicular lymphoma. Management of grade 3A follicular lymphoma remains controversial and treatment should be individualized; while grades 1-2 are typically managed as indolent disease and grade 3B is treated analogously to diffuse large B-cell lymphoma (DLBCL), grade 3A may be approached with either strategy depending on clinical context [8,19,20].

DOTATATE avidity in inflammatory conditions, mediated by SSTR expression on activated lymphocytes and proinflammatory macrophages, has been documented yet remains uncommon [[19], [20], [21], [22]]. Both Crohn’s disease and lymphoma can produce bowel wall thickening and mucosal ulceration, complicating interpretation. In this case, the deep ulcerations and cobblestoning favored Crohn’s disease, while the multifocal nature of the small bowel involvement and the absence of typical Crohn's complications added diagnostic uncertainty [[23], [24], [25]]. Notably, an IBD serologic panel was negative, highlighting the atypical nature of her presentation and the limitations of serologic testing in establishing this diagnosis. Diagnosis of Crohn's disease ultimately rests on the combination of endoscopic, histologic, and radiographic findings rather than any single test [26]. Treatment typically involves biologic agents such as anti-TNF inhibitors or integrin inhibitors for moderate-to-severe disease [26]. In this patient, initiation of Crohn's-directed therapy was deferred pending response to lymphoma treatment given the complexity of managing concurrent immunosuppression.

The L4 vertebral lesion exhibited characteristic CT features of vertebral hemangioma and remained stable on serial imaging over 18 months. Vertebral hemangiomas are common benign vascular tumors and present incidentally in up to 26% of the population on spine imaging [27]. DOTATATE uptake in vertebral hemangiomas has been documented as a recognized false-positive finding, attributed to vascular endothelial SSTR expression [28]. As hemangiomas are typically asymptomatic and require no treatment, correlation with CT findings is necessary to distinguish them from metastatic disease [4,28,29].

The co-occurrence of follicular lymphoma, Crohn's disease, and vertebral hemangioma, 3 distinct pathologies each capable of somatostatin receptor expression, in a single patient represents a rare but instructive diagnostic scenario. This case illustrates that DOTATATE positivity should not reflexively confirm a neuroendocrine tumor diagnosis when clinical context and anatomic imaging features are discordant, as the breadth of potential false-positive etiologies remains underappreciated in practice. Tissue confirmation is essential when imaging findings are atypical, and careful correlation with anatomic imaging, clinical history, and laboratory findings is necessary to avoid misdiagnosis.

## Conclusion

This case illustrates the rare co-occurrence of 3 distinct SSTR-expressing pathologies, follicular lymphoma, Crohn disease, and vertebral hemangioma, exhibiting ^68^Ga-DOTATATE uptake on PET/CT in a pattern mimicking metastatic neuroendocrine tumor. It underscores that accurate interpretation of DOTATATE imaging requires integration of metabolic findings with anatomic imaging, clinical history, and laboratory data. When imaging or clinical features are discordant with a NET, tissue confirmation remains essential to avoid misattribution of DOTATATE uptake to neuroendocrine malignancy.

## Patient consent

Complete written informed consent was obtained from the patient for the publication of this study and accompanying images.
